# LJM716 in Japanese patients with head and neck squamous cell carcinoma or HER2-overexpressing breast or gastric cancer

**DOI:** 10.1007/s00280-016-3214-4

**Published:** 2016-12-09

**Authors:** Shunji Takahashi, Takayuki Kobayashi, Junichi Tomomatsu, Yoshinori Ito, Hisanobu Oda, Tatsuhiro Kajitani, Tomoyuki Kakizume, Takeshi Tajima, Hiromi Takeuchi, Heiko Maacke, Taito Esaki

**Affiliations:** 1 Department of Medical Oncology, The Cancer Institute Hospital of Japanese Foundation for Cancer Research, 3-8-31 Ariake, Koto-ku, Tokyo, 135-8550 Japan; 2 Department of Breast Medical Oncology, The Cancer Institute Hospital of Japanese Foundation for Cancer Research, Tokyo, Japan; 3 Department of Gastrointestinal and Medical Oncology, National Hospital Organization Kyushu Cancer Center, Fukuoka, Japan; 4 Novartis Pharma K.K., Tokyo, Japan; 5 Saiseikai Fukuoka General Hospital, Fukuoka, Japan

**Keywords:** HER3, HER2, LJM716, Monoclonal antibody, Phase I

## Abstract

**Purpose:**

Human epidermal growth factor receptor 3 (HER3) has been identified as an important component of many receptor tyrosine kinase-driven cancers. LJM716 is a human IgG monoclonal antibody that binds HER3, trapping it in an inactive conformation. In this study, a phase I dose escalation was performed with a primary objective to establish the maximum tolerated dose and/or the recommended dose of LJM716 in Japanese patients with selected advanced solid tumors. Secondary objectives included the evaluation of the safety and tolerability, preliminary antitumor activity, and pharmacokinetics of LJM716 in Japanese patients.

**Methods:**

LJM716 was administered intravenously at doses of 10, 20, or 40 mg/kg once weekly, in 28-day cycles, to 12 patients with HER2-amplified breast cancer or gastric cancer, or with esophageal squamous cell carcinoma or squamous cell carcinoma of the head and neck, regardless of HER2 status.

**Results:**

The maximum tolerated dose was not reached, and the recommended dose was established at 40 mg/kg. No dose-limiting toxicities were observed in the first cycle. The most frequently reported adverse events were diarrhea, fatigue, stomatitis, pyrexia, and paronychia. One unconfirmed partial response was observed in a patient with breast cancer, and 50% of the patients achieved stable disease as the best overall response. Exposure increased with ascending dose, and half-life was estimated to be 11–14 days. No anti-LJM716 antibodies were detected.

**Conclusions:**

LJM716 was well tolerated in Japanese patients, and a degree of tumor shrinkage was observed.

**Clinical trial information:**

ClinicalTrials.gov NCT01911936.

**Electronic supplementary material:**

The online version of this article (doi:10.1007/s00280-016-3214-4) contains supplementary material, which is available to authorized users.

## Introduction

Inappropriate activation of the human epidermal growth factor receptor (HER) family of tyrosine kinases has been implicated in a wide variety of different cancers [1]. Activation occurs via homo- or heterodimerization of HER family members, inducing kinase activity and subsequent downstream activation of intracellular signaling pathways. HER3 lacks significant kinase activity [2] but has recently been recognized as an important component of receptor tyrosine kinase-driven tumorigenesis, dimerizing with and activating other HER family members, such as epidermal growth factor receptor (EGFR) and HER2 [3]. Overexpression of HER2 or interaction with the ligand, neuroregulin1 (NRG1), promotes HER2:HER3 heterodimerization and subsequent phosphoinositide 3-kinase signaling, driving tumor cell proliferation and tumor growth [1, 3]. HER3 therefore plays an important role in the development and maintenance of HER2- and NRG1-driven cancers, and represents an attractive target for directed therapy.

The efficacy of HER2-directed therapies has already been demonstrated, and approved drugs include the monoclonal antibodies, trastuzumab and pertuzumab, and the tyrosine kinase inhibitor, lapatinib [4–6]. Although the use of these drugs has vastly improved the treatment options for patients with HER2-driven cancer, problems with resistance have been documented. Resistance can develop through multiple mechanisms, including cross-activation by and compensatory upregulation of other HER family members, including HER3 [7]. As such, targeting HER2 and HER3 simultaneously is hoped to improve response to treatment; indeed, promising outcomes have been reported following combination of HER2-directed therapies with other therapies [8–10]. A number of cancer types are good candidates for HER3-directed therapy. Dysregulated HER2 expression has been documented in esophageal squamous cell carcinoma (ESCC; 31%) [11], metastatic breast cancer (18–20%) [7], and gastric/gastroesophageal junction cancer (15–30%) [12]. In addition to HER2-driven cancers, preclinical data suggest that NRG1-driven cancers, which include a significant proportion of squamous cell carcinomas of the head and neck (SCCHN), are also likely to benefit from HER3-directed therapy [13, 14].

LJM716 is a fully human IgG monoclonal antibody that specifically binds HER3, trapping it in the inactive conformation. LJM716 is uniquely able to block both the ligand-independent and ligand-dependent modes of HER3 activation, and antitumor activity has been demonstrated in both HER2-amplified and NRG1-driven xenograft models [15, 16]. In a recent global phase I clinical trial in predominantly Western Caucasian patients with HER2-overexpressing breast cancer or gastric cancer, and with ESCC or SCCHN, regardless of HER2 status (NCT01598077), single-agent LJM716 was well tolerated up to a recommended dose (RD) of 40 mg/kg [17]. Here, the safety and tolerability of single-agent LJM716 were evaluated in Japanese patients in the same indications.

## Materials and methods

### Study oversight

This was a phase I, open-label, multicenter study (clinicaltrials.gov registry number NCT01911936) [18]. The accrual period, from the first patient visit to the last patient visit, was from September 19, 2013, to March 6, 2015. The study was designed by the sponsor (Novartis Pharmaceuticals Corporation) and performed in accordance with the principles of Good Clinical Practice. The protocol was approved by an Institutional Review Board at each institution, and the study was conducted according to the ethical principles of the Declaration of Helsinki. All patients provided written informed consent before any study procedures.

### Patient selection

All patients were aged ≥18 years, had an Eastern Cooperative Oncology Group (ECOG) performance status of ≤2, and had HER2-overexpressing or amplified (HER2+) locally advanced/metastatic breast cancer or gastric cancer, or recurrent or metastatic SCCHN or ESCC, regardless of HER2 status, for which no effective treatment option exists (investigator decision). For breast cancer, patients were required to have tumors with 3+ HER2-overexpression documented by immunohistochemistry or amplification by in situ hybridization; for gastric cancer (including gastroesophageal junction tumors), patients were required to have tumors with immunohistochemistry 2+ or 3+ and amplification by in situ hybridization [19, 20]. Exclusion criteria included patients with untreated or symptomatic central nervous system metastases, other primary malignancies requiring intervention, or prior treatment with an anti-HER3 antibody.

### Study objectives

The primary objectives for the study were to determine the maximum tolerated dose (MTD) and/or RD of LJM716 as a single agent when administered intravenously (IV) to Japanese patients with advanced solid tumors. The secondary objectives were to characterize the safety and tolerability, pharmacokinetics (PK), and preliminary antitumor activity of LJM716, and to assess the emergence of antibodies against LJM716.

### Study design and treatment plan

During the dose-escalation part, at least 12 patients were planned to be treated in successive cohorts. The starting dose was 10 mg/kg LJM716, followed by 20 and 40 mg/kg, given by IV infusion over 2 h once weekly (QW) in 28-day cycles. An adaptive Bayesian logistic regression model (BLRM) [21] incorporating escalation with overdose control criteria was used to guide dose-escalation decisions [22, 23], and provide support to establish the MTD or RD for LJM716.

Premedication prior to each dose of LJM716 was recommended (650 mg acetaminophen or equivalent and 50 mg IV diphenhydramine or equivalent) to circumvent infusion-related reactions. Dose reductions to ≥10 mg/kg were permitted, as were dose interruptions ≤28 days. LJM716 administration was discontinued in the event of disease progression, unacceptable adverse events (AEs), or as the result of patient or physician decision. It was decided not to open the dose-expansion part of this study.

### Toxicity assessments

Safety assessments were carried out based on all AEs and their relationship to the study drug treatment, with regular monitoring of hematology, blood chemistry, and urine analysis, and regular assessment of vital signs, physical condition, body weight, performance status, and cardiac function. AEs were assessed according to the National Cancer Institute Common Terminology Criteria for Adverse Events, version 4.03. Dose-limiting toxicities (DLTs) were defined as AEs or abnormal laboratory values assessed as unrelated to disease progression, intercurrent illness, or concomitant medication, as defined in Supplemental Table 1.

### Response assessments

Tumor lesions were assessed by investigators according to Response Evaluation Criteria In Solid Tumors (RECIST) guidelines, version 1.1 [24]. Patients underwent screening computed tomography (CT) scans of the chest, abdomen, and pelvis, and MRI where evaluation by CT was not adequate. Visible skin lesions and easily palpable subcutaneous tumors were measured by physical examination. Screening was performed within 28 days of the first dose. The post-baseline RECIST assessments were performed every two cycles and then at the end of treatment if a scan was not conducted 30 days prior to this.

### Pharmacokinetics and immunogenicity

Serum was collected for PK assessments at multiple time points, including serial samplings to calculate PK parameters (Cycle 1 and Cycle 3) and trough samplings (Day 1 of each cycle). The serum concentration of LJM716 was measured using a validated ELISA. PK parameters (maximum observed serum concentration after drug administration [*C*_max_], time to* C*_max_ [*T*_max_], area under the curve [AUC], etc.) were determined by a non-compartmental method with a lower limit of quantification of 150 ng/mL. Serum immunogenicity was assessed using an anti-LJM716 antibody test.

## Results

### Patient characteristics

A total of 12 patients were treated with intravenous LJM716 at doses of 10, 20, and 40 mg/kg QW, between September 19, 2013, and March 6, 2015. The median age of patients was 58 years (range, 33‒75); 8/12 (67%) patients were aged <65 years; 6/12 (50%) were male; 4/12 (33%) had an ECOG performance status of 1; and 1/12 (8%) had an ECOG performance status of 2. Confirmed primary tumor types at enrollment were ESCC [*n* = 2 (17%)], SCCHN [*n* = 2 (17%)], HER2-overexpressing breast cancer [*n* = 6 (50%)], and HER2-overexpressing gastric cancer [*n* = 2 (17%)]. All patients had stage IV disease at study entry. All patients had received prior antineoplastic therapies (median 6; range, 2–13), including trastuzumab in eight patients. Patient demographics are given in Table 1. The median duration of exposure was 14.0 weeks (range, 4.0–48.1) across all LJM716 doses, and most patients (92%) had an exposure of >4 weeks (Supplemental Table 2). All 12 patients discontinued treatment due to disease progression.

**Table 1:** Patient demographics and disease characteristics, by treatment group

	10 mg/kg QW (*n* = 3)	20 mg/kg QW (*n* = 3)	40 mg/kg QW (*n* = 6)	All patients (*N* = 12)
Age, years (median)	69.0	58.0	51.5	58.0
<65, *n* (%)	1 (33)	2 (67)	5 (83)	8 (67)
≥65, *n* (%)	2 (67)	1 (33)	1 (17)	4 (33)
Sex, *n* (%)				
Female	2 (67)	1 (33)	3 (50)	6 (50)
Male	1 (33)	2 (67)	3 (50)	6 (50)
ECOG PS, *n* (%)				
0	1 (33)	1 (33)	5 (83)	7 (58)
1	2 (67)	2 (67)	0	4 (33)
2	0	0	1 (17)	1 (8)
Primary site of cancer, *n* (%)				
Breast	2 (67)	1 (33)	3 (50)	6 (50)
Esophagus	0	0	2 (33)	2 (17)
Head and neck	0	1 (33)	1 (17)	2 (17)
Gastric	1 (33)	1 (33)	0	2 (17)
Primary tumor histology, *n* (%)				
Squamous cell carcinoma	0	1 (33)	3 (50)	4 (33)
Adenocarcinoma	3 (100)	2 (67)	3 (50)	8 (67)
Stage at study entry, *n* (%)				
IV	2 (67)	2 (67)	5 (83)	9 (75)
IVB	1 (33)	1 (33)	1 (17)	3 (25)

*ECOG* Eastern Cooperative Oncology Group, *PS* performance status, *QW* once weekly

### Toxicity

No DLTs were reported in the first cycle of the study. Grade 3 pneumonia aspiration observed in one patient (40 mg/kg) during Cycle 2 met the DLT criteria as described in Supplemental Table 1 (other ≥grade 3 non-hematologic toxicity). The MTD was not reached, and the RD was established at 40 mg/kg QW based on the BLRM, safety, tolerability, and PK data. All patients had at least one AE, regardless of study drug relationship. The most frequent AEs in all patients were diarrhea (50%), stomatitis (42%), fatigue, edema peripheral, and pyrexia (33% each). There were no clinically relevant differences in AEs across the study groups. At the RD of 40 mg/kg, the most frequent AEs were diarrhea, pyrexia (50% each), fatigue, nasopharyngitis, anemia, and lymphocyte count decreased (33% each; Table 2). AEs assessed as infusion-related reactions were only observed in the 40 mg/kg dose group (pyrexia in three patients and headache in one patient). Five grade 3/4 AEs were reported: pneumonia aspiration, anemia, neutropenia, hyponatremia, and hypophosphatemia in one patient (8%) each, and decreased lymphocyte count in two patients (17%). Ten patients (83%) had AEs suspected to be study drug-related, and the most common was diarrhea (50%; Supplemental Table 3). Two patients (17%) experienced grade 3/4 AEs suspected to be study drug-related: pneumonia aspiration and neutropenia in one patient (8%) each, and decreased lymphocyte count in two patients (17%). Two patients reported serious AEs: nausea and vomiting, and pneumonia aspiration in one patient (8%) each, of which only pneumonia aspiration was suspected to be study drug-related. No deaths were reported during the study. No AEs that led to study drug discontinuation were reported, and four patients (33%) reported AEs requiring dose interruption: influenza, atrial fibrillation, neutropenia, nasopharyngitis, and pneumonia aspiration in one patient each. No AEs leading to dose reduction were reported.

**Table 2:** Adverse events (≥10%), regardless of study drug relationship, by treatment group

Preferred term, *n* (%)	10 mg/kg QW (*n* = 3)	20 mg/kg QW (*n* = 3)	40 mg/kg QW (*n* = 6)	All patients (*N* = 12)
Diarrhea	2 (67)	1 (33)	3 (50)	6 (50)
Stomatitis	3 (100)	1 (33)	1 (17)	5 (42)
Fatigue	1 (33)	1 (33)	2 (33)	4 (33)
Edema peripheral	1 (33)	2 (67)	1 (17)	4 (33)
Pyrexia	0	1 (33)	3 (50)	4 (33)
Nausea	1 (33)	1 (33)	1 (17)	3 (25)
Cough	2 (67)	0	1 (17)	3 (25)
Pruritus	1 (33)	1 (33)	1 (17)	3 (25)
Nasopharyngitis	0	1 (33)	2 (33)	3 (25)
Paronychia	2 (67)	0	1 (17)	3 (25)
Anemia	0	1 (33)	2 (33)	3 (25)
Decreased appetite	1 (33)	1 (33)	1 (17)	3 (25)
Dysphagia	0	1 (33)	1 (17)	2 (17)
Vomiting	1 (33)	1 (33)	0	2 (17)
Dyspnea	0	1 (33)	1 (17)	2 (17)
Rash	1 (33)	0	1 (17)	2 (17)
Headache	1 (33)	0	1 (17)	2 (17)
Peripheral sensory neuropathy	0	1 (33)	1 (17)	2 (17)
Lymphocyte count decreased	0	0	2 (33)	2 (17)

*QW* once weekly

### Efficacy

**Fig. 1:**
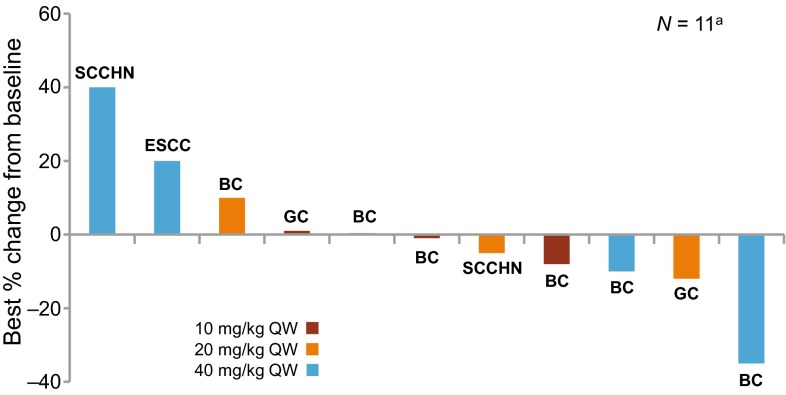
Best percentage change from baseline in target lesions by treatment group. *BC* breast cancer, *ESCC* esophageal squamous cell carcinoma, *GC* gastric cancer, *QW* once weekly, *SCCHN* squamous cell carcinoma of the head and neck. ^a^The number of patients was 11 because one patient with ESCC did not have target lesions

Of all treated patients, six (50%) achieved stable disease and six (50%) had progressive disease (Table 3). None of the patients achieved complete or partial response; however, 4/6 patients with HER2+ breast cancer showed some tumor shrinkage (Fig. 1). One patient with HER2+ breast cancer (40 mg/kg) had an unconfirmed partial response (tumor shrinkage of more than 30% in Cycle 10), followed by subsequent progressive disease.

**Table 3:** Best overall response in all disease types (investigator assessed)

Best overall response, *n* (%)	10 mg/kg QW (*n* = 3)	20 mg/kg QW (*n* = 3)	40 mg/kg QW (*n* = 6)	All patients (*N* = 12)
Complete response	0	0	0	0
Partial response	0	0	0	0
Stable disease	3 (100)	1 (33)	2 (33)	6 (50)
Unconfirmed CR/PR	0	0	1 (17)	1 (8)
Progressive disease	0	2 (67)	4 (67)	6 (50)
Unknown	0	0	0	0

Data cut off: March 6, 2015

*CR* complete response, *PR* partial response, *QW* once weekly

### Pharmacokinetic studies and immunogenicity

The PK parameters for Cycle 1 and Cycle 3 are given in Table 4, and these were comparable to those seen previously in a global study in predominantly Western Caucasian patients [17]. The exposure of LJM716 increased as the dose increased. Upon a power model analysis of the dose–exposure relationship for AUC from time zero to the time of the last quantifiable concentration (AUC_0–last_) and* C*_max_ in Cycle 1, dose-proportionality in the dose range 10–40 mg/kg was suggested with slope estimates of 0.82 (90% confidence interval [CI] 0.53–1.12) and 0.85 (90% CI 0.65–1.04), respectively, near to unity. There was 2.8–3.4-fold accumulation at steady state in Cycle 3 after repeated weekly doses, based on AUC after the first doses of Cycles 1 and 3. The effective half-life of LJM716 was estimated to be 11–14 days, based on the accumulation. All patients were tested for antidrug antibodies against LJM716, but antibodies were not detected in any of the samples.

**Table 4:** Pharmacokinetic parameters for LJM716 (Cycle 1; Cycle 3)

	AUC_0–last_ (h·μg/mL)	*C*_min_ (μg/mL)	*C*_max_ (μg/mL)	*T*_max_ (h)
*Cycle 1*	*Mean (SD)*			*Median (range)*
10 mg/kg (*n* = 3)	18,700 (7100)	65.8 (27.3)	195 (53.9)	4.38 (2.83‒9.57)
20 mg/kg (*n* = 3)	33,700 (5120)	137 (27.8)	362 (53.4)	4.63 (2.07‒9.65)
40 mg/kg (*n* = 6)	59,000 (21,500)	243 (100)	628 (136)	3.75 (2.02‒9.65)
*Cycle 3* ^a^	*Individual data*			
10 mg/kg (*n* = 2)	35,300; 62,400	185; 280	268; 615	9.50; 9.53
40 mg/kg (*n* = 1)	243,000	1210	2130	4.73

*AUC*_0–last_ area under the curve from time zero to the time of the last quantifiable concentration, *C*_max_ maximum observed serum concentration after drug administration, *C*_min_ minimum drug serum concentration, SD standard deviation, *T*_max_ time to reach* C*_max_

^a^The number of available patients was limited in Cycle 3

## Discussion

This study evaluated the safety and tolerability of IV-administered LJM716 in Japanese patients with solid tumors. The RD of LJM716 was established at 40 mg/kg QW; the same as that determined in a global clinical trial in Western Caucasian patients [17]. The MTD was not reached during the course of this study. LJM716 was well tolerated with a manageable safety profile, with observed toxicities largely grade 1 or 2. The most frequently observed AE was diarrhea, which is seen with most other HER family inhibitors [25–27]. Diarrhea in this study was mild. Similarly, skin toxicities were generally less frequent and milder than seen with other HER family inhibitors [6, 28]. The fact that these toxicities were mild (grade 1 or 2) might be considered to be due to the specificity of LJM716 for HER3; more severe AEs could be expected to result from off-target effects. Similarly, more severe AEs are seen in combination studies, with grade 3 gastrointestinal problems and skin toxicities being reported in trials combining HER2 or HER3-targeted mAbs with EGFR inhibitors [29–34]. Of 12 patients treated with LJM716, only one serious AE suspected to be drug induced was reported. This was aspiration-induced pneumonia in a patient with SCCHN. While the cause for this is unknown, serious bacterial pneumonia has been reported in two other studies with HER3 mAbs [27, 33]. Overall, LJM716 was found to have a favorable safety profile.

LJM716 demonstrated dose-dependent exposure, with an estimated half-life of 11–14 days, and PK parameters similar to those observed in the global study and in line with other therapeutic antibodies. The AUC/*C*_max_ observed at the RD was sufficient to achieve effective systemic drug levels, based on preclinical studies. In the preclinical study, 1–10 nM LJM716 suppressed growth of HER2-overexpressing or NRG-stimulated cell lines [15]. In this study, the mean* C*_max_ was 243 μg/mL (equivalent to ~1–2 μM based on a typical molecular weight of ~150 kDa for a human IgG mAb) for the 40 mg/kg dose group at Cycle 1, and significantly higher at Cycle 3.

Although no confirmed complete or partial responses were observed during the course of the study, tumor shrinkage of more than 30% (unconfirmed partial response) was observed in one patient with HER2+ breast cancer (40 mg/kg group), and tumor shrinkage was also observed in three of the other five patients with HER2+ breast cancer across all doses. A total of 50% of the patients achieved stable disease. Five of the six patients with HER2+ breast cancer showing stable disease or an unconfirmed partial response had documented progression with the most recent prior regimen, which contained trastuzumab, and the sixth patient received trastuzumab as part of the second most recent treatment regimen. Further investigation is needed to confirm whether LJM716 will be an efficacious treatment option for patients with breast cancer who are trastuzumab resistant.

Future development of HER3-directed therapies may benefit from the consideration of appropriate biomarkers. NRG1 has been validated as a predictive biomarker for response to the HER3-directed mAbs AV-203 and patritumab in preclinical and in clinical studies, respectively [35, 36]. In contrast, no correlation was seen between tumor inhibition and HER3 levels [35].

Although the single-agent LJM716 antitumor activity observed here was less extensive than that observed in preclinical xenograft studies, these same studies established that efficacy was significantly improved by combination of LJM716 with other HER2-directed therapies [15, 16]. Consistent with this, there is an increasing body of clinical evidence demonstrating that the efficacy of HER2/HER3-directed therapies is improved by combination with other anti-HER antibodies [9, 37], tyrosine kinase inhibitors [33, 34], and/or chemotherapy [8, 10]. For example, in a phase II trial in which 100% of the patients progressed on pertuzumab single-agent therapy, a clinical benefit rate (stable disease + partial response + complete response ≥6 months) of 41% was achieved following subsequent combination therapy with trastuzumab [37]. It is noteworthy that the addition of LJM716 to trastuzumab resulted in increased inhibition of pAKT and improved in vitro efficacy exceeding that achieved by the combination of trastuzumab with pertuzumab [15]. Based on these observations, the future development of LJM716 is likely to focus on combination with other therapies, and a phase I study of LJM716 in combination with trastuzumab in patients with HER2-overexpressing breast or gastric cancer is currently ongoing (NCT01602406).

## Electronic supplementary material

Below is the link to the electronic supplementary material.

Supplementary material 1 (PDF 287 kb)
