# Supplementary material for: LJM716 in Japanese patients with head and neck squamous cell carcinoma or HER2-overexpressing breast or gastric cancer
Source: Cancer Chemother Pharmacol. 2016 Dec 9;79(1):131–8. doi: 10.1007/s00280-016-3214-4 (PMC5225197; doi:10.1007/s00280-016-3214-4)
Supplement: Supplementary file 1 — Supplementary material 1 (PDF 287 kb) [file 280_2016_3214_MOESM1_ESM.pdf]

LJM716 in Japanese patients with head and neck squamous cell carcinoma or HER2-overexpressing breast or gastric cancer

Cancer Chemotherapy and Pharmacology

Shunji Takahashi,[1] Takayuki Kobayashi,[1] Junichi Tomomatsu,[1] Yoshinori Ito,[2] Hisanobu Oda,[3†] Tatsuhiro Kajitani,[3] Tomoyuki Kakizume,[4] Takeshi Tajima,[4] Hiromi Takeuchi,[4] Heiko Maacke[4] and Taito Esaki[3]

[1]Department of Medical Oncology, The Cancer Institute Hospital of Japanese Foundation for Cancer Research, Tokyo, Japan; [2]Department of Breast Medical Oncology, The Cancer Institute Hospital of Japanese Foundation for Cancer Research, Tokyo, Japan; [3]Department of Gastrointestinal and Medical Oncology, National Hospital Organization Kyushu Cancer Center, Fukuoka, Japan; [4]Novartis Pharma K.K., Tokyo, Japan

Email: s.takahashi-chemotherapy@jfcr.or.jp

Supplemental Table 1 Summary of criteria for dose-limiting toxicities (CTCAE version 4.03 grading)

DLTs are specified for various organs not limited to hematology, renal, hepatic, cardiac, and pulmonary toxicities
Hematologic
≥ grade 3 neutropenia for >7 consecutive days, ≥ grade 3 (with clinical bleeding) or grade 4 thrombocytopenia, grade 4 anemia, febrile neutropenia
Laboratory renal/hepatic
Serum creatinine >2 x ULN; ≥ grade 3 total bilirubin increased (or grade 2 with grade 3 ALT increased), grade 4 ALT increased (or grade 3 for >7 consecutive days for patients without liver metastases)
Cardiac and pulmonary
≥ grade 2 heart failure or ≥ grade 3 cardiac disorder; ≥ grade 3 (or grade 2 lasting >7 days in same cycle) pulmonary disorder
Other
Other ≥ grade 3 non-hematologic toxicity or investigator-assessed unacceptable toxicity considered to be dose limiting
Exceptions
<72 hours of CTCAE grade 3 fatigue; alopecia, inadequately treated nausea, vomiting, or diarrhea; clinically non-significant, treatable, or reversible laboratory abnormalities

ALT alanine aminotransferase, *AST* aspartate aminotransferase, *CTCAE* Common Terminology Criteria for Adverse Events, *DLTs* dose-limiting toxicities, *ULN* upper limit of normal

Supplemental Table 2 Duration of exposure to drug by treatment group

Duration of exposure	10 mg/kg QW (*n* = 3)	20 mg/kg QW (*n* = 3)	40 mg/kg QW (*n* = 6)	All patients (*N* = 12)
Median	22	7	8	14
(range)	(20–24)	(4–32)	(6–48)	(4–48)
Duration of exposure (weeks), *n* (%)				
1–4	0	1 (33)	0	1 (8)
>4–8	0	1 (33)	4 (67)	5 (42)
>16	3 (100)	1 (33)	2 (33)	6 (50)

QW once weekly

Supplemental Table 3 Adverse events (≥10 %), suspected to be related to study treatment, by treatment group

Preferred term, *n* (%)	10 mg/kg QW (*n* = 3)	20 mg/kg QW (*n* = 3)	40 mg/kg QW (*n* = 6)	All patients (*N* = 12)
Diarrhea	2 (67)	1 (33)	3 (50)	6 (50)
Stomatitis	3 (100)	0	0	3 (25)
Fatigue	1 (33)	1 (33)	1 (17)	3 (25)
Pyrexia	0	0	3 (50)	3 (25)
Paronychia	2 (67)	0	1 (17)	3 (25)
Nausea	1 (33)	0	1 (17)	2 (17)
Edema peripheral	1 (33)	0	1 (17)	2 (17)
Pruritus	1 (33)	0	1 (17)	2 (17)
Rash	1 (33)	0	1 (17)	2 (17)
Peripheral sensory neuropathy	0	1 (33)	1 (17)	2 (17)
Cough	1 (33)	0	1 (17)	2 (17)
Anemia	0	0	2 (33)	2 (17)
Lymphocyte count decreased	0	0	2 (33)	2 (17)

QW once weekly
